# Association between wooden toy engagement and cognitive function among Chinese older adults: a cross-sectional study

**DOI:** 10.3389/fpsyg.2025.1546657

**Published:** 2025-05-08

**Authors:** Hanqian Wang, Qiuping Cheng, Ping Mei, Mengni Cui, Qunlong Wang, Lu Li

**Affiliations:** ^1^Institute of Social Medicine, School of Medicine, Zhejiang University, Hangzhou, Zhejiang, China; ^2^Zhejiang Shuren University, Hangzhou, Zhejiang, China; ^3^Fuyun Yuanhe Street Community Health Service Center, Lishui, Zhejiang, China; ^4^College of Food Engineering, East University of Heilongjiang, Haerbin, Heilongjiang, China; ^5^Hangzhou International Urbanology Research Center & Zhejiang Urban Governance Studies Center, Hangzhou, China

**Keywords:** older adults, cognitive function, wooden toys, social entertainment, cross-sectional study

## Abstract

**Background:**

The rising prevalence of cognitive impairment among older adults poses a significant public health challenge. While wooden toys are traditionally regarded as tools for cognitive stimulation, research on their potential relationship with cognitive function in older adults remains limited. This study aims to explore the association between engagement with wooden toys and cognitive function in older adults.

**Methods:**

A cross-sectional study was conducted in May 2023, involving 387 older adults aged 60–94 years from Yunhe County, Zhejiang Province, China. Both bivariate analysis and multivariable linear regression analysis were performed to assess the association between wooden toy engagement and cognitive function.

**Results:**

Among the participants, 22.2% reported having experience with wooden toys. Older adults who played with wooden toys regularly or occasionally demonstrated significantly better cognitive performance compared to those who had never played. Importantly, the association between toy engagement frequency and cognitive function varied across age groups. Specifically, adults aged 60–64 who engaged with wooden toys regularly scored higher on the cognitive function than those who did not. Similarly, participants aged 65–69 who played regularly or occasionally also exhibited superior cognitive scores. Multivariable linear regression analysis confirmed that participation in wooden toy activities was positively associated with cognitive function (*B* = 0.86, 95% CI: 0.14–1.57 in the MMSE model; *B* = 0.99, 95% CI: 0.16–1.83 in the MoCA model).

**Conclusion:**

This study highlights a positive association between engagement with wooden toys and cognitive function in older adults. The frequency of engagement emerged as a critical factor in this relationship, with higher levels of participation linked to better cognitive outcomes. These findings offer valuable insights for developing strategies and policies to prevent or slow cognitive decline in aging populations. Promoting simple, accessible activities such as playing with wooden toys may serve as an effective intervention to enhance cognitive health among older adults.

## Introduction

Cognitive impairment, a precursor to dementia, represents a growing public health concern with substantial societal and economic burdens (Bradburn et al., 2019). Globally, approximately 15.56% of the population suffers from mild cognitive impairment (MCI) (Bai et al., 2022), a condition that significantly elevates the risk of developing dementia later in life (Bradburn et al., 2019). In China, 15.5% of adults aged 60 and older are affected by MCI, equivalent to nearly 38.8 million individuals (Jia et al., 2020), underscoring the urgent need for focused attention on this issue.

Demographic variables and lifestyle behaviors are well-documented risk factors for cognitive decline. Advanced age, lower levels of education, and a history of vascular disease are consistently linked to increased risk (Lee et al., 2020). Additionally, lifestyle factors such as smoking, alcohol consumption, physical activity, and diet have been shown to influence cognitive health (Rosenberg et al., 2018). Recent studies have emphasized the importance of cognitive engagement, with activities such as intellectual pursuits (Cheng et al., 2014), mental exercises, and brain games (Xue et al., 2021) demonstrating protective effects against cognitive decline. These activities, including reading, chess, and card games, have been associated with enhanced cognitive function and a lower incidence of dementia in elderly populations (Yates et al., 2016; Litwin et al., 2017; Wang et al., 2012). In China, traditional games like mahjong, puzzles, and card-playing have been highlighted for their potential cognitive benefits, drawing interest for their widespread use among older adults (Tian et al., 2022; Wang et al., 2022). Studies on cognitive stimulation from activities like mahjong have shown better eye–hand coordination (Tsang et al., 2016) and strategic thinking and experience a sense of being socially connected (Teh and Tey, 2019), with mahjong associated with better cognitive flexibility and social interaction (Tse et al., 2024). Puzzles, on the other hand, have also been linked to improvements in spatial reasoning and memory (Fissler et al., 2018; Gelfo et al., 2018).

Beyond intellectual games, sensory and social activities also contribute to cognitive well-being. Wooden toys, in particular, offer a unique blend of cognitive stimulation, emotional comfort, and social interaction, factors crucial for cognitive health and mental health in older adults (CGTN, 2021; Gao et al., 2020; Yunhe News, 2022). These toys provide opportunities for both individual and cooperative play, fostering engagement independent of venue and offering diverse options tailored to individual preferences and capabilities. Research has shown that engaging in self-directed activities, such as those facilitated by wooden toys, can enhance autonomy and improve mental health outcomes by increasing a sense of control and self-determination (Menec, 2003). Increasing individuals’ freedom and autonomy in decision-making can boost their participation in activities, leading to improved self-motivation and better mental health outcomes (Ryan and Deci, 2000). Despite growing interest and the global development of numerous educational wooden toys for their potential to stimulate mental faculties and enhance cognitive health in older adults (Murray et al., 2003), the potential cognitive benefits of wooden toys remain underexplored. One recent cluster-randomized trial (Cheng et al., 2024) has examined the efficacy of wooden toy interventions on cognitive decline among older adults with mild cognitive impairment, reporting promising results in a nursing home setting. Another study conducted in a nursing institution (Cui et al., 2025) has also shown that after the intervention of wooden-toys training, the enrichment and diversity of gut microbiome in the older adults with cognitive impairment was significantly higher than that in the baseline stage as their cognitive function improved. However, the absence of community-based data calls for further investigation to validate these findings across broader settings.

The role of other intellectual activities in mitigating cognitive decline has been extensively studied (Tian et al., 2022; Tsang et al., 2016; Teh and Tey, 2019; Tse et al., 2024; Fissler et al., 2018; Gelfo et al., 2018; Chu-Man et al., 2015; Perrot and Maillot, 2023), and these activities seem to share some common benefits with wooden toys in terms of cognitive stimulation. However, they differ in the ways they engage older adults in cognitive processing. For instance, puzzles require individual problem-solving (Fissler et al., 2018), while mahjong involves social interaction and strategic thinking (Tse et al., 2024). Wooden toys may have a positive impact on stimulating imagination, enhancing fine motor skills, and fostering creativity, but the specific mechanisms remain unclear, and even little is known about their specific effects on cognitive function.

This study aims to address this gap by investigating the relationship between engagement with wooden toys and cognitive performance in a community-based survey of older adults. We hypothesize that frequent engagement with wooden toys is associated with better cognitive performance, particularly in domains of executive function, visuospatial skills, orientation, and attention.

## Methods

### Participants

Data for this study come from a cross-sectional study conducted in Yunhe County, Zhejiang Province, China, in May 2023. Based on a previous study (Yang et al., 2016) in Zhejiang, the prevalence (p) of cognitive impairment was estimated at 22.0%. Using a permissible error of 0.2p, the required sample size was calculated to be 341 participants. Considering the non-response rate and refusal rate based on previous studies, we further expanded our sample by 10% and came to a theoretical sample size of 376. Stratified random sampling was adopted to recruit study participants from Yunhe County. Initially, two communities and two administrative villages were randomly selected from urban and rural areas. Subsequently, two community apartments and two villages were randomly chosen from each of these areas, resulting in a total of four community apartments and four villages included in the study. Adults aged 60 years or older who were able to provide written informed consent were invited to participate in the study. A total of 389 older adults completed the questionnaire, while two (0.5%) questionnaires were excluded due to inconsistencies in responses to logical validation questions. Consequently, 387 valid questionnaires were included in the analysis. This study was approved by the Ethics Review Board of Zhejiang Shuren University (2023SK030), and all the participants have provided written informed consent.

### Measures

#### Sociodemographic and health-related characteristics

Sociodemographic characteristics included in the analysis were participants’ age, gender, education level, marital status, monthly income, residence, and occupation before retirement. Education levels were categorized into illiterate, primary school, middle school, and high school and above. Marital status was divided into married and otherwise. Occupation before retirement included professional and technical personnel, governmental, institutional, or managerial personnel, commercial service or industrial workers, agriculture, forestry, animal husbandry or fishery workers, houseworkers, and so on. To assess their health status, participants were asked whether they had any chronic diseases and were further asked to self-report their health status. The self-reported health status was classified into excellent, good, fair, poor, and very poor. Additionally, a self-reported assessment to identify depression in older individuals was performed using the 15-item Geriatric Depression Scale (GDS-15) (Zhang et al., 2022). The total score of the GDS-15 is calculated as the sum of the 15 items, with a higher score indicating more depressive symptoms.

#### Wooden toy engagement

The wooden toys included in this study were designed to stimulate various cognitive functions and motor skills. Games such as “Pitching Pot” and “Ring Toss” were designed to enhance fine motor skills, while “Tangram” and “Huarong Path Slide Puzzle” aimed to stimulate logical reasoning and problem-solving abilities. Additionally, tools like the “Tower of Hanoi” and “Memory Chess” were used to promote memory retention (Cheng et al., 2024). These activities could be performed individually, in pairs, or in group settings, allowing for a range of engagement opportunities.

The frequency of engagement with wooden toys was recorded by asking participants how often they played with these toys in their daily lives. There were no restrictions on the types of wooden toys participants could report using. Based on the frequency of play, participants in our study were classified into the “regularly” group, the “occasionally” group, and the “never” group. In the study, the “regularly” group included participants who, at the time of asking, played with wooden toys at least once a week, either on a daily basis or nearly every day. The “occasionally” group consisted of those who, at the time of asking, played with wooden toys at least once a month but less than weekly, or occasionally but not monthly. The “never” group comprised participants who, at the time of asking, never engaging with wooden toys.

#### Cognitive function

The Chinese versions of the Montreal Cognitive Assessment (MoCA-BC) together with the Mini-mental State Examination (MMSE) were used to assess the cognitive function of older adults in this study. The MoCA and MMSE have been extensively used in previous studies and have been proven to have good validity and reliability (Chen et al., 2016; Creavin et al., 2016; Koski, 2013). In addition, the MoCA test was sensitive to mild cognitive impairment, whereas the MMSE was used primarily to screen out dementia (Creavin et al., 2016; Koski, 2013). There are five subsections in MMSE (orientation, registration, attention and calculation, recall, and language) and six subsections in MoCA (executive functioning, attention, language, visuo-spatial, orientation, and memory) (Aiello et al., 2022). For both tests, the potential scores ranged from 0 to 30, with higher values indicating better cognition.

### Statistical analysis

Continuous variables were presented as mean ± standard deviations (SD), while categorical variables were expressed as frequency and percentage. Chi-square (*χ*^2^) tests were used to identify the group differences for categorical variables. To examine the relationship between engagement with wooden toys and cognitive function (MMSE and MoCA), one-way ANOVA was conducted, followed by Bonferroni post-hoc tests for multiple comparisons, stratified by age. Multivariable linear regression analysis was used to identify factors associated with cognitive function. Variables found to be statistically significant in the chi-square tests were included in the regression model. The primary independent variable was the frequency of playing with wooden toys, while cognitive function was assessed using the MMSE and MoCA scores as dependent variables. All statistical analyses were performed using SPSS 21.0, with a significance threshold set at *p* < 0.05.

## Results

### Participant characteristics

The study included 387 participants with a mean age of 68.3 years (SD = 5.98), ranging from 60 to 94 years (Table 1). More than half of the participants were female (54.0%), and resided in rural areas (57.9%). Among all the participants, 80.9% reported being married, 35.7% had a primary school education, and 46.3% reported a monthly income between 2000 and 3,999 yuan. Additionally, 41.9% of participants had worked in agriculture, forestry, animal husbandry, or fishery before the age of 60. Most of the older adults (71.6%) reported no diagnosis of chronic diseases, and 53.2% assessed their health status as fair. The mean scores for GDS-15, MMSE, and MoCA of all the participants were 6.41 ± 1.83, 23.5 ± 5.02, and 21.74 ± 5.91, respectively.

**Table 1:** Characteristics of the study participants (*n* = 387).

Variables	Full sample (*n* = 387) *N*(%)	Playing with wooden toys			*p*
		Regularly (*n* = 45)	Occasionally (*n* = 41)	Never (*n* = 301)	
Age group, years					<0.001
60–64	112 (28.9)	18 (40.0)	21 (51.2)	73 (24.3)	
65–69	136 (35.1)	23 (51.1)	13 (31.7)	100 (33.2)	
> = 70	139 (35.9)	4 (8.9)	7 (17.1)	128 (42.5)	
Gender					0.009
Male	178 (46.0)	28 (62.2)	12 (29.3)	138 (45.8)	
Female	209 (54.0)	17 (37.8)	29 (70.7)	163 (54.2)	
Education level					<0.001
Illiterate	42 (10.9)	0 (0)	1 (2.4)	41 (13.6)	
Primary school	138 (35.7)	7 (15.6)	11 (26.8)	120 (39.9)	
Middle school	93 (24.0)	7 (15.6)	10 (24.4)	76 (25.2)	
High school and above	114 (29.5)	31 (68.9)	19 (46.3)	64 (21.3)	
Marital status					0.005
Married	313 (80.9)	41 (91.1)	39 (95.1)	233 (77.4)	
Otherwise	74 (19.1)	4 (8.9)	2 (4.9)	68 (22.6)	
Monthly income					<0.001
<2000 yuan ($281)	90 (23.3)	0 (0)	11 (26.8)	79 (26.2)	
2000–3,999 yuan ($281–562)	179 (46.3)	13 (28.9)	13 (31.7)	153 (50.8)	
4,000–5,999 yuan ($563–843)	59 (15.2)	13 (28.9)	8 (19.5)	38 (12.6)	
6,000–7,999 yuan ($843–1,124)	46 (11.9)	16 (35.6)	5 (12.2)	25 (8.3)	
≥8,000 yuan ($1,125)	13 (3.4)	3 (6.7)	4 (9.8)	6 (2.0)	
Residence					<0.001
Rural	224 (57.9)	10 (22.2)	19 (46.3)	195 (64.8)	
Urban	163 (42.1)	35 (77.8)	22 (53.7)	106 (35.2)	
Occupation before retirement					<0.001
Professional and technical personnel	45 (11.6)	12 (26.7)	4 (9.8)	29 (9.6)	
Governmental, institutional, or managerial personnel	47 (12.1)	18 (40.0)	12 (29.3)	17 (5.6)	
Commercial, service, or industrial worker	46 (11.9)	3 (6.7)	6 (14.6)	37 (12.3)	
Agriculture, forestry, animal husbandry, or fishery worker	162 (41.9)	6 (13.3)	12 (29.3)	144 (47.8)	
Houseworker	43 (11.1)	0 (0)	3 (7.3)	40 (13.3)	
Other	44 (11.4)	6 (13.3)	4 (9.8)	34 (11.3)	
Chronic diseases					0.005
Yes	110 (28.4)	22 (48.9)	11 (26.8)	77 (25.6)	
No	277(71.6)	23 (51.1)	30 (73.2)	224 (74.4)	
Self-rated Health					0.001
Excellent	37 (9.6)	9 (20.0)	6 (14.6)	22 (7.3)	
Good	127 (32.8)	21 (46.7)	19 (46.3)	87 (28.9)	
Fair	206 (53.2)	15 (33.3)	16 (39.0)	206 (53.2)	
Poor	16 (4.1)	0 (0)	0 (0)	16 (4.1)	
Very poor	1 (0.3)	0 (0)	0 (0)	1 (0.3)	
GDS-15 score, M(SD)	6.42 (1.83)	6.40 (1.83)	6.59 (2.28)	6.40 (1.76)	0.822
MMSE score, M(SD)	23.50 (5.02)	26.33 (3.30)	26.12 (2.91)	22.71 (5.19)	<0.001
MoCA score, M(SD)	21.74 (5.91)	25.24 (3.91)	24.51 (3.52)	20.84 (6.11)	<0.001

### Engaging with wooden toys

In this study, 11.6% of older adults reported regularly playing with wooden toys, 10.6% reported occasional use, and 77.8% reported never playing with these wooden toys. Older adults who played with wooden toys regularly were more likely to be male (62.2% vs. 37.8%, *p* = 0.009), aged 65–69 years (*p* < 0.001), better educated (*p* < 0.001), married (91.1% vs. 8.9%, *p* = 0.005), and had a higher average monthly income (*p* < 0.001). The “regularly” group were also more likely to reside in urban areas (77.8% vs. 22.2%, *p* < 0.001), have worked as governmental, institutional, or managerial personnel (*p* < 0.001), report no chronic disease diagnoses (51.1% vs. 48.9%, *p* = 0.005), and rate their health as good (*p* < 0.001). The “regularly” group demonstrated significantly better performance on the MMSE (26.33 vs. 26.12 vs. 22.71, *p* < 0.01) and MoCA (25.24 vs. 24.51 vs. 20.84, *p* < 0.001) scores compared to the “occasionally” and “never” groups. However, no significant differences were found among the three groups with concerning GDS-15 scores.

### The association between playing with wooden toys and cognitive function

**Figure 1:**
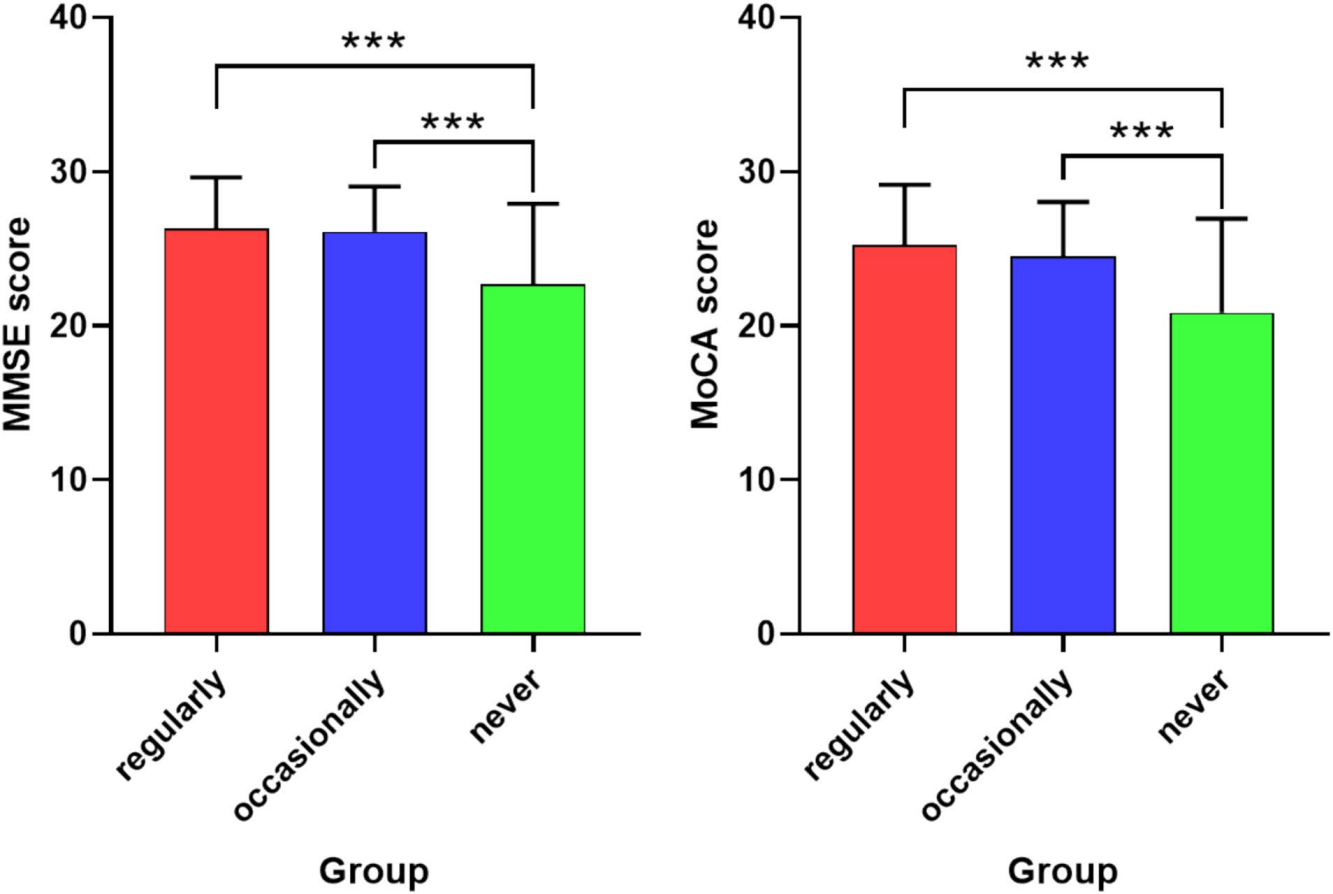
Multiple comparisons of cognitive function among older adults with different frequencies of playing with wooden toys. The error bars reflect the standard errors of the means. *** represents *p* < 0.001; ** represents *p* < 0.01; and * represents *p* < 0.05.

Figure 1 shows the relationship between playing with wooden toys and overall cognitive function scores. Statistically significant differences were observed in both the MMSE and MoCA scores across the three groups. Multiple comparisons revealed that there were differences in MMSE and MoCA scores between the “regularly” and “never” groups, with the “regularly” group having significantly higher MMSE and MoCA scores than the “never” group. Similarly, the “occasionally” group also had significantly higher MMSE and MoCA scores compared to the “never” group.

Table 2 presents the relationship between playing with wooden toys and the MMSE and MoCA dimensions for all participants. Statistically significant differences were found across all MMSE dimensions, including orientation, registration, attention and calculation, recall, and language (all *p-*values <0.01) among the three different groups. Likewise, all MoCA dimensions, including executive functioning, attention, language, visuo-spatial, orientation, and memory, showed statistically significant differences (all *p-*values <0.01) among the three groups. Overall, participants who regularly or occasionally played with wooden toys performed better in all dimensions of cognitive function compared to those who never played.

**Table 2:** The relationship between playing with wooden toys and MMSE items and MoCA items.

Variables	Playing with wooden toys			*F*	*p*
	Regularly	Occasionally	Never		
MMSE score	26.33 (3.30)	26.12 (2.91)	22.71 (5.19)	17.88	<0.001
Orientation	9.91 (0.47)	9.90 (0.37)	9.32 (1.25)	9.24	<0.001
Registration	2.24 (0.96)	2.34 (0.82)	1.70 (1.15)	9.80	<0.001
Attention and calculation	4.09 (1.38)	4.12 (1.44)	3.40 (1.79)	5.71	0.004
Recall	1.67 (1.21)	1.41 (1.05)	0.99 (0.97)	10.75	<0.001
Language	8.42 (0.81)	8.34 (0.88)	7.31 (1.77)	14.80	<0.001
MoCA score	25.24 (3.91)	24.51 (3.52)	20.84 (6.11)	17.30	<0.001
Executive Functioning	3.07 (1.01)	2.44 (1.18)	2.13 (1.29)	11.54	<0.001
Attention	5.42 (1.01)	5.32 (1.11)	4.51 (1.75)	9.45	<0.001
Language	4.67 (0.71)	4.73 (0.55)	3.88 (1.34)	15.19	<0.001
Visuo-spatial	3.29 (1.01)	3.27 (0.90)	2.79 (1.27)	5.57	0.004
Orientation	5.96 (0.30)	5.95 (0.22)	5.50 (0.89)	11.02	<0.001
Memory	2.84 (1.76)	2.80 (1.33)	2.04 (1.53)	8.74	<0.001

The relationships between playing with wooden toys and MMSE and MoCA scores stratified by age are described in Table 3. For participants aged 60–64, there was a significant difference in total MMSE score across the three groups (*p* = 0.004). The “regularly” group scored significantly higher than the “never” group on the MMSE score (*p* < 0.05). For participants aged 65–69 years old, statistically significant differences were observed in the total MMSE score among the three groups (*p* = 0.004). *Post hoc* analyses indicated that both the “regularly” group and “occasionally” group had higher MMSE scores (*p* < 0.05) compared to the “never” group. Regarding the MoCA score, there were significant differences among the three different groups for participants aged 60–64 years (*p* = 0.011) and 65–69 years (*p* = 0.004). For participants aged 60–64 years old, the “never” group had lower MoCA scores than the “regularly” group (*p* < 0.05). For participants aged 65–69 years old, both the “regularly” group and “occasionally” group had higher MoCA scores than the “never group” (*p* < 0.05). No significant difference was found in cognitive function scores between the “regularly” and “occasionally” groups.

**Table 3:** The relationship between playing with wooden toys and MMSE score and MoCA score in older adults stratified by age.

Variables	Playing with wooden toys			*F*	*p*	Post-hoc
	Regularly (1)	Occasionally (2)	Never (3)			
MMSE score						
Participants aged 60–64 years old	27.50 (2.60)	25.95 (2.69)	24.41 (4.08)	5.71	0.004	1 > 3**
Participants aged 65–69 years old	25.78 (3.58)	26.54 (2.50)	23.08 (4.98)	5.66	0.004	1 > 3*, 2 > 3*
Participants aged > = 70 years old	24.25 (3.30)	25.86 (4.38)	21.46 (5.62)	2.51	0.085	
MoCA score						
Participants aged 60–64 years old	26.22 (2.71)	23.95 (3.44)	22.84 (4.71)	4.69	0.011	1 > 3**
Participants aged 65–69 years old	25.00 (4.42)	25.23 (3.00)	21.29 (5.82)	6.51	0.002	1 > 3**, 2 > 3*
Participants aged > = 70 years old	22.25 (4.57)	24.86 (4.78)	19.34 (6.66)	2.67	0.073	

Data are n/N (%) or mean (standard deviation, SD), unless otherwise indicated.

Post hoc indicates the significance of pairwise comparisons in the post-hoc analysis using Bonferroni tests.

*** represents *p* < 0.001; ** represents *p* < 0.01; and * represents *p* < 0.05.

Table 4 displays the results of multivariable linear regression analyses examining the relationship between MMSE and MoCA score, playing with wooden toys, and other covariates. Older age was significantly associated with lower MMSE and MoCA scores (*B* = −1.12, *p* < 0.001; *B* = −1.27, *p* < 0.001, respectively). Residence was also found to be associated with both MMSE and MoCA scores (*B* = 2.61, *p* < 0.001; *B* = 3.52, *p* < 0.001, respectively), with older adults residing in urban areas exhibiting better cognitive performance. Playing with wooden toys was positively associated with both MMSE and MoCA scores (*B* = 0.86, *p* = 0.019; *B* = 0.99, *p* = 0.020, respectively). More frequent engagement with wooden toys was linked to higher cognitive function scores and better overall cognitive performance.

**Table 4:** Multivariable linear regression analysis of the association between MMSE scores and MoCA scores and playing with wooden toys.

	*B*	SE	*p*	95%CI	*F*
MMSE					16.27***
Age	−1.12	0.30	<0.001	−1.71, −0.52	
Gender	−0.71	0.47	0.135	−1.64, 0.22	
Marital status	−0.68	0.62	0.273	−1.89, 0.54	
Residence	2.61	0.56	<0.001	1.51, 3.70	
Occupation before retirement	−0.25	0.19	0.210	−0.62, 0.14	
Chronic diseases	0.95	0.52	0.069	−0.07, 1.97	
Playing with wooden toys	0.86	0.36	0.019	0.14, 1.57	
MoCA					17.45***
Age	−1.27	0.35	<0.001	−1.96, −0.57	
Gender	−0.88	0.55	0.113	−1.97, 0.21	
Marital status	−0.71	0.72	0.326	−2.12, 0.71	
Residence	3.52	0.65	<0.001	2.24, 4.80	
Occupation before retirement	−0.29	0.22	0.204	−0.73, 0.16	
Chronic diseases	0.62	0.61	0.309	−0.57, 1.81	
Playing with wooden toys	0.99	0.42	0.020	0.16, 1.83	

B = Unstandardized Coefficients in the regression; SE = standard errors; CI = confidence interval.

*** represents *p* < 0.001; ** represents *p* < 0.01; and * represents *p* < 0.05.

## Discussion

Our study found that older adults who engaged regularly or occasionally with wooden toys had better cognitive function compared to those who did not participate in these activities. Significant differences were observed in both the MMSE and MoCA scores, including multiple subdomains, between the “regularly” and “never” groups. Notably, older adults aged 60 to 69 who participated in these activities showed significantly better cognitive performance than their peers who did not engage in such activities.

Our linear regression analysis revealed that playing with wooden toys was associated with better cognitive function in older adults, even after controlling for covariates such as sociodemographic factors. These findings support our initial hypothesis, showing that older adults who engage with these toys have cognitive advantages over those who do not. This relationship suggests a complex link between wooden toys and cognitive function in older adults, offering a novel and engaging way for them to maintain or enhance cognitive performance.

The benefits of engaging with wooden toys for cognitive health are clear. Individuals who regularly or occasionally engage with these toys showed improved performance in various cognitive domains. Specifically, they demonstrate better skills in MMSE areas such as orientation, registration, attention and calculation, memory, and language. These areas are essential components of cognitive function, particularly in older adults, and are often used to assess early signs of cognitive decline. Additionally, these individuals also perform better in MoCA assessments, which include executive function, attention, language, visuospatial abilities, orientation, and memory. These improvements in both the MMSE and MoCA scores highlight the positive impact of engaging in activities that stimulate both the mind and body. This finding aligns with the results of a meta-analysis (Perrot and Maillot, 2023), which suggests that exercise-based games—those that integrate physical movement with cognitive challenges—are particularly beneficial for maintaining and enhancing cognitive health in older adults. These types of activities not only stimulate the brain but also promote physical health, creating a holistic approach to maintaining cognitive function. Further buttressing this notion, Murray et al. (2003). supported this view, observing that exposure to various forms of play, including toys, led to improved cognitive, language, and emotional abilities in Alzheimer’s patients. This underscores the therapeutic potential of toys in enhancing both mental and emotional well-being, particularly for individuals with cognitive impairments like Alzheimer’s disease. In line with these findings, a randomized trial (Xue et al., 2021) substantiated that structured game training can positively recalibrate the cognitive spectrum of older adults. Other studies (Chu-Man et al., 2015; Cheng et al., 2014) have also shown that participation in these activities or training can preserve cognitive function or delay decline in certain cognitive domains, even in those with significant cognitive impairment. This suggests that even for those with pre-existing cognitive challenges, such activities may offer a meaningful way to sustain cognitive health and improve quality of life.

It is compelling to note that the relationship between engagement with wooden toys and cognitive function is not universally consistent, but rather varies across different age groups. Notably, our study found that individuals aged 65–69 who regularly or occasionally interacted with these toys had significantly higher MMSE and MoCA scores compared to those who did not engage with the toys. Similarly, the 60–64 age group showed superior cognitive performance when they frequently played with the toys, outperforming their non-participating counterparts. This pattern suggests that the frequency of engagement may play a critical role in modulating the cognitive benefits derived from these activities. These findings are consistent with previous research, such as studies by Wang et al. (2022) and Zhou et al. (2020), which have highlighted a frequency-dependent relationship between cognitive health and engagement in activities like card-playing. Likewise, longitudinal observational studies have shown that more frequent participation in leisure activities, such as playing cards or mahjong, is associated with a significant reduction in the risk of cognitive impairment among older adults (Mao et al., 2020; Kurita et al., 2020; Qiu et al., 2019).

In evaluating the factors associated with cognitive function, our study highlighted age, residence, and engagement with wooden toys as key contributors. While age and residence are fixed determinants, engagement with wooden toys represents a modifiable factor that can be positioned as a potential intervention for enhancing cognitive function in older adults.

Although the underlying mechanisms of the effects of wooden toys on cognitive function in older adults remain somewhat unclear, our observations suggest several potential pathways through which these toys may influence cognitive function. One possible mechanism is that wooden toys promote both physical and cognitive stimulation. For instance, smaller educational toys such as tangrams challenge cognitive processes such as problem-solving, spatial reasoning, and memory. Larger wooden toys, which require more physical engagement, may stimulate fine motor skills, hand-eye coordination, and physical activity (Murray et al., 2003), which are all linked to improvements in cognitive function (Bherer, 2015). These toys may also foster social interactions, providing emotional support, and reducing stress, all of which have been shown to positively impact cognitive health (Zhu et al., 2016). These mechanisms are consistent with the Activity Theory (McAvinia, 2016) and findings from studies on similar activities (Teh and Tey, 2019; Tse et al., 2024), where physical activity and social interaction were found to significantly improve cognitive outcomes in older adults. Moreover, these toys may serve as tools for cognitive training, offering a personalized approach to mental and emotional well-being (Cheng et al., 2024). By engaging with wooden toys tailored to an individual’s cognitive needs, older adults can experience more targeted cognitive stimulation. This aligns with findings from research conducted in nursing home (Cheng et al., 2024), where personalized engagement with toys was found to have a positive impact on both cognitive function and emotional well-being.

Despite these insightful findings, our study has some limitations. Wooden toys are a relatively novel intervention among older adults, and additional research with larger and more diverse samples would be valuable to confirm our findings. The cross-sectional design prevents us from drawing causal conclusions about the relationship between wooden toys use and cognitive function. While individuals with cognitive decline may reduce their engagement in cognitive activities, our study emphasizes the potential positive impact of such activities on cognitive function, which could inform future interventions. A more robust longitudinal study or a well-designed randomized controlled trial would strengthen our conclusions. Additionally, the small sample size limits our exploration of the various types of wooden toys and their specific effects on cognitive function. Different toy types may have unique effects on cognition, and further investigation is needed to understand these distinctions. Our study also did not account for several potential confounding variables, such as other cognitive and physical activities, lifestyle factors, and mood, which may influence cognitive outcomes. Future research should control for these variables to clarify the relationship between wooden toy engagement and cognitive function. Moreover, subgroup analyses of participants aged ≥70 years, particularly those who engage with wooden toys regularly or occasionally, were limited by small sample sizes. However, the effect sizes for MMSE (*η*^2^ = 0.0356) and MoCA (*η*^2^ = 0.0377) suggest meaningful results. Larger and more representative samples are needed to validate and generalize these findings. Given the limited direct comparisons between wooden toys and other cognitively stimulating activities, future research should consider a more systematic investigation of these different forms of play. Comparative studies could help clarify the unique cognitive benefits of wooden toys and better understand how they contribute to older adults relative to other activities like puzzles and mahjong.

## Conclusion

Our study provides evidence of an association between engagement with wooden toys and cognitive function in Chinese older adults. The findings suggest that more frequent engagement with wooden toys may play a significant role in reducing the risk of cognitive decline in this demographic. This study adds to the growing body of knowledge about the relationship between cognitive decline and leisure activities, highlighting the potential benefits of engaging with wooden toys as a novel recreational activity. Wooden toys, serving not only as a source of entertainment but also as a means of providing emotional comfort and promoting social interaction, can be considered a promising intervention strategy for mitigating cognitive decline among older adults. By emphasizing the value of such simple and accessible activities, this research underscores the importance of incorporating recreational tools like wooden toys into broader cognitive health and wellness programs for older adults.

## Data Availability

The datasets used and analyzed during the current study are available from the corresponding author on reasonable request. Requests to access the datasets should be directed to Qunlong Wang, zjsrujzyl@126.com.
